# Works in Progress

**Published:** 1843-06

**Authors:** 


### Works in Progress.

*New Work on Dental Surgery.*—Carey & Hart, have in preparation a work on the *Anatomy,* Physiology, and Pathology of the teeth, including Practical Dentistry, by Dr. Goddard, with numerous and beautiful engravings in illustration of the text. [*Bulletin of Medical Science.*

---

*Regnum Animale, by Swedenborg.*—An English translation of this great physiological work, we learn from the British Quarterly Journal of Dental Surgery, is soon to be published by Mr. Newbery, of London. It will also be published by Mr. Otis Clapp, of School street, Boston.

